# Supplementary material for: Development of chemokine network inhibitors using combinatorial saturation mutagenesis
Source: Commun Biol. 2025 Apr 3;8:549. doi: 10.1038/s42003-025-07778-6 (PMC11969024; doi:10.1038/s42003-025-07778-6)
Supplement: Supplementary file 4 — Supplementary Data 2 [file 42003_2025_7778_MOESM4_ESM.zip › SupplementaryData3/scripts/xattune_ci.tar!/./script/analysis/JK/JK_REPS_ExactPvalues.docx]

Supplementary Table: Exact P values for cell migration experiments, comparisons
to control

| ID | Experiment components | Control | Number of biological replicates | P value |
| --- | --- | --- | --- | --- |
| R13 | THP1 | HD2 | 4 | 0.00e+00 |
| R13 | THP1,CCL14 | HD2 | 4 | 9.98e-01 |
| R13 | THP1,CCL14,HD2 | HD2 | 4 | NA |
| R13 | THP1,CCL14,HD2SCR | HD2 | 4 | 1.00e+00 |
| R13 | THP1,CCL14,HD2Y5W | HD2 | 3 | 9.79e-01 |
| R13 | THP1,CCL14,HD2T6W | HD2 | 3 | 1.00e+00 |
| R13 | THP1,CCL14,HD2A9W | HD2 | 3 | 1.00e+00 |
| R13 | THP1,CCL14,HD2T16C | HD2 | 3 | 2.76e-03 |
| R15 | THP1 | HD2 | 3 | 1.00e-06 |
| R15 | THP1,CCL23 | HD2 | 3 | 1.00e+00 |
| R15 | THP1,CCL23,HD2 | HD2 | 3 | NA |
| R15 | THP1,CCL23,HD2SCR | HD2 | 3 | 8.55e-01 |
| R15 | THP1,CCL23,HD2Y5W | HD2 | 3 | 1.47e-01 |
| R15 | THP1,CCL23,HD2T6W | HD2 | 3 | 5.87e-01 |
| R15 | THP1,CCL23,HD2A9W | HD2 | 3 | 1.55e-02 |
| R15 | THP1,CCL23,HD2T16C | HD2 | 3 | 2.55e-02 |
| R16 | J:CXCR1 | HD2 | 3 | 2.50e-05 |
| R16 | J:CXCR1,CXCL6 | HD2 | 3 | 1.00e-05 |
| R16 | J:CXCR1,CXCL6,HD2 | HD2 | 3 | NA |
| R16 | J:CXCR1,CXCL6,HD2SCR | HD2 | 3 | 2.12e-02 |
| R16 | J:CXCR1,CXCL6,HD2Y5W | HD2 | 3 | 2.06e-01 |
| R16 | J:CXCR1,CXCL6,HD2T6W | HD2 | 3 | 2.36e-04 |
| R16 | J:CXCR1,CXCL6,HD2T6D | HD2 | 3 | 1.00e+00 |
| R16 | J:CXCR1,CXCL6,HD2A9W | HD2 | 3 | 2.47e-04 |
| R16 | J:CXCR1,CXCL6,HD2L11I | HD2 | 3 | 1.00e+00 |
| R16 | J:CXCR1,CXCL6,HD2T16C | HD2 | 3 | 9.20e-05 |
| R20 | ATC | HD2 | 5 | 9.79e-03 |
| R20 | ATC,CCL19 | HD2 | 5 | 4.85e-02 |
| R20 | ATC,CCL19,HD2 | HD2 | 5 | NA |
| R20 | ATC,CCL19,HD2SCR | HD2 | 5 | 9.99e-01 |
| R20 | ATC,CCL19,HD2Y5W | HD2 | 3 | 1.78e-02 |
| R20 | ATC,CCL19,HD2T6W | HD2 | 3 | 1.55e-02 |
| R20 | ATC,CCL19,HD2A9W | HD2 | 3 | 2.16e-02 |
| R20 | ATC,CCL19,HD2T16C | HD2 | 3 | 1.31e-02 |
| R21 | ATC | HD2 | 4 | 4.19e-03 |
| R21 | ATC,CXCL11 | HD2 | 4 | 8.92e-02 |
| R21 | ATC,CXCL11,HD2 | HD2 | 4 | NA |
| R21 | ATC,CXCL11,HD2SCR | HD2 | 4 | 1.49e-01 |
| R21 | ATC,CXCL11,HD2Y5W | HD2 | 4 | 1.18e-02 |
| R21 | ATC,CXCL11,HD2T6W | HD2 | 4 | 6.63e-04 |
| R21 | ATC,CXCL11,HD2A9W | HD2 | 4 | 5.73e-04 |
| R21 | ATC,CXCL11,HD2T16C | HD2 | 3 | 1.45e-03 |
| R25 | THP1 | HD2 | 3 | 2.37e-01 |
| R25 | THP1,CCL5 | HD2 | 3 | 0.00e+00 |
| R25 | THP1,CCL5,HD2 | HD2 | 3 | NA |
| R25 | THP1,CCL5,HD2SCR | HD2 | 3 | 0.00e+00 |
| R25 | THP1,CCL5,HD2Y5W | HD2 | 3 | 1.32e-01 |
| R25 | THP1,CCL5,HD2T6W | HD2 | 3 | 1.00e+00 |
| R25 | THP1,CCL5,HD2A9W | HD2 | 3 | 9.95e-01 |
| R25 | THP1,CCL5,HD2T16C | HD2 | 3 | 1.00e-05 |
| R26 | THP1 | HD2 | 3 | 1.45e-02 |
| R26 | THP1,CCL7 | HD2 | 3 | 4.03e-04 |
| R26 | THP1,CCL7,HD2 | HD2 | 3 | NA |
| R26 | THP1,CCL7,HD2SCR | HD2 | 3 | 3.22e-04 |
| R26 | THP1,CCL7,HD2Y5W | HD2 | 3 | 6.66e-01 |
| R26 | THP1,CCL7,HD2T6W | HD2 | 3 | 8.87e-01 |
| R26 | THP1,CCL7,HD2A9W | HD2 | 3 | 7.33e-01 |
| R26 | THP1,CCL7,HD2T16C | HD2 | 3 | 1.00e+00 |
| R27 | THP1 | HD2 | 3 | 1.00e+00 |
| R27 | THP1,CCL8 | HD2 | 3 | 0.00e+00 |
| R27 | THP1,CCL8,HD2 | HD2 | 3 | NA |
| R27 | THP1,CCL8,HD2SCR | HD2 | 3 | 0.00e+00 |
| R27 | THP1,CCL8,HD2Y5W | HD2 | 3 | 8.86e-01 |
| R27 | THP1,CCL8,HD2T6W | HD2 | 3 | 9.45e-01 |
| R27 | THP1,CCL8,HD2A9W | HD2 | 3 | 9.12e-01 |
| R27 | THP1,CCL8,HD2T16C | HD2 | 3 | 9.61e-01 |
| R44 | ATC | HD2 | 5 | 0.00e+00 |
| R44 | ATC,CXCL9 | HD2 | 5 | 3.86e-02 |
| R44 | ATC,CXCL9,HD2 | HD2 | 5 | NA |
| R44 | ATC,CXCL9,HD2SCR | HD2 | 5 | 9.24e-01 |
| R44 | ATC,CXCL9,HD2Y5W | HD2 | 3 | 0.00e+00 |
| R44 | ATC,CXCL9,HD2T6W | HD2 | 3 | 9.00e-06 |
| R44 | ATC,CXCL9,HD2T6D | HD2 | 3 | 1.00e+00 |
| R44 | ATC,CXCL9,HD2A9W | HD2 | 3 | 4.79e-02 |
| R44 | ATC,CXCL9,HD2L11I | HD2 | 3 | 4.00e-06 |
| R44 | ATC,CXCL9,HD2T12W | HD2 | 3 | 4.14e-03 |
| R44 | ATC,CXCL9,HD2T16C | HD2 | 3 | 0.00e+00 |
| R45 | ATC | HD2 | 3 | 3.00e-06 |
| R45 | ATC,CCL21 | HD2 | 3 | 9.61e-01 |
| R45 | ATC,CCL21,HD2 | HD2 | 3 | NA |
| R45 | ATC,CCL21,HD2SCR | HD2 | 3 | 1.00e+00 |
| R45 | ATC,CCL21,HD2Y5W | HD2 | 3 | 5.40e-05 |
| R45 | ATC,CCL21,HD2T6W | HD2 | 3 | 8.87e-02 |
| R45 | ATC,CCL21,HD2T6D | HD2 | 3 | 9.96e-01 |
| R45 | ATC,CCL21,HD2A9W | HD2 | 3 | 1.07e-01 |
| R45 | ATC,CCL21,HD2L11I | HD2 | 3 | 4.29e-01 |
| R45 | ATC,CCL21,HD2T12W | HD2 | 3 | 8.36e-01 |
| R45 | ATC,CCL21,HD2T16C | HD2 | 3 | 4.00e-06 |
| R47 | ATC | HD2Y5W | 6 | 0.00e+00 |
| R47 | ATC,CXCL12 | HD2Y5W | 6 | 0.00e+00 |
| R47 | ATC,CXCL12,HD2Y5W | HD2Y5W | 6 | NA |
| R47 | ATC,CXCL12,HD2SCR | HD2Y5W | 6 | 2.00e-06 |
| R47 | ATC,CXCL12,HD2 | HD2Y5W | 6 | 7.00e-06 |
| R47 | ATC,CXCL12,HD2T6W | HD2Y5W | 3 | 9.99e-01 |
| R47 | ATC,CXCL12,HD2T6D | HD2Y5W | 3 | 2.17e-01 |
| R47 | ATC,CXCL12,HD2L11I | HD2Y5W | 3 | 1.54e-02 |
| R47 | ATC,CXCL12,HD2T12W | HD2Y5W | 3 | 9.95e-01 |
| R47 | ATC,CXCL12,HD2T16C | HD2Y5W | 3 | 1.48e-04 |
| R47 | ATC,CXCL12,HD2CM304 | HD2Y5W | 3 | 2.34e-04 |
| R47 | ATC,CXCL12,HD2CM307 | HD2Y5W | 3 | 9.99e-01 |
| R47 | ATC,CXCL12,HD2CM452 | HD2Y5W | 3 | 9.12e-04 |
| R47 | ATC,CXCL12,HD2CM526 | HD2Y5W | 3 | 1.00e+00 |
| R47 | ATC,CXCL12,HD2CM539 | HD2Y5W | 3 | 2.15e-01 |
| R61 | THP1 | HD2Y5W | 3 | 0.00e+00 |
| R61 | THP1,CCL2 | HD2Y5W | 3 | 7.77e-01 |
| R61 | THP1,CCL2,HD2Y5W | HD2Y5W | 3 | NA |
| R61 | THP1,CCL2,HD2SCR | HD2Y5W | 3 | 4.60e-01 |
| R61 | THP1,CCL2,HD2 | HD2Y5W | 3 | 1.00e+00 |
| R61 | THP1,CCL2,HD2T6W | HD2Y5W | 3 | 1.00e+00 |
| R61 | THP1,CCL2,HD2T6D | HD2Y5W | 3 | 1.00e+00 |
| R61 | THP1,CCL2,HD2A9W | HD2Y5W | 3 | 1.00e+00 |
| R61 | THP1,CCL2,HD2L11I | HD2Y5W | 3 | 1.00e+00 |
| R61 | THP1,CCL2,HD2T12W | HD2Y5W | 3 | 9.98e-01 |
| R61 | THP1,CCL2,HD2T16C | HD2Y5W | 3 | 7.79e-01 |
| R61 | THP1,CCL2,HD2CM304 | HD2Y5W | 3 | 1.00e+00 |
| R61 | THP1,CCL2,HD2CM307 | HD2Y5W | 3 | 1.11e-01 |
| R61 | THP1,CCL2,HD2CM452 | HD2Y5W | 3 | 9.79e-01 |
| R61 | THP1,CCL2,HD2CM526 | HD2Y5W | 3 | 1.00e+00 |
| R61 | THP1,CCL2,HD2CM539 | HD2Y5W | 3 | 7.82e-01 |
| R62 | ATC | HD2Y5W | 3 | 4.00e-06 |
| R62 | ATC,CXCL11 | HD2Y5W | 3 | 3.50e-01 |
| R62 | ATC,CXCL11,HD2Y5W | HD2Y5W | 3 | NA |
| R62 | ATC,CXCL11,HD2SCR | HD2Y5W | 3 | 9.28e-01 |
| R62 | ATC,CXCL11,HD2 | HD2Y5W | 3 | 4.47e-01 |
| R62 | ATC,CXCL11,HD2T6W | HD2Y5W | 3 | 8.23e-01 |
| R62 | ATC,CXCL11,HD2T6D | HD2Y5W | 3 | 1.00e+00 |
| R62 | ATC,CXCL11,HD2L11I | HD2Y5W | 3 | 1.00e+00 |
| R62 | ATC,CXCL11,HD2T12W | HD2Y5W | 3 | 9.97e-01 |
| R62 | ATC,CXCL11,HD2T16C | HD2Y5W | 3 | 3.72e-02 |
| R62 | ATC,CXCL11,HD2CM304 | HD2Y5W | 3 | 2.17e-03 |
| R62 | ATC,CXCL11,HD2CM452 | HD2Y5W | 3 | 3.99e-02 |
| R62 | ATC,CXCL11,HD2CM539 | HD2Y5W | 3 | 1.46e-04 |
| R64 | ATC | HD2Y5W | 3 | 1.00e+00 |
| R64 | ATC,CCL21 | HD2Y5W | 3 | 3.00e-06 |
| R64 | ATC,CCL21,HD2Y5W | HD2Y5W | 3 | NA |
| R64 | ATC,CCL21,HD2SCR | HD2Y5W | 3 | 9.00e-05 |
| R64 | ATC,CCL21,HD2 | HD2Y5W | 3 | 8.50e-05 |
| R64 | ATC,CCL21,HD2T6W | HD2Y5W | 3 | 5.21e-01 |
| R64 | ATC,CCL21,HD2T6D | HD2Y5W | 3 | 2.98e-04 |
| R64 | ATC,CCL21,HD2L11I | HD2Y5W | 3 | 2.08e-02 |
| R64 | ATC,CCL21,HD2T12W | HD2Y5W | 3 | 2.87e-03 |
| R64 | ATC,CCL21,HD2CM304 | HD2Y5W | 3 | 9.99e-01 |
| R64 | ATC,CCL21,HD2CM452 | HD2Y5W | 3 | 9.87e-01 |
| R66 | THP1 | HD2Y5W | 4 | 1.29e-02 |
| R66 | THP1,CCL3 | HD2Y5W | 4 | 1.00e+00 |
| R66 | THP1,CCL3,HD2Y5W | HD2Y5W | 4 | NA |
| R66 | THP1,CCL3,HD2SCR | HD2Y5W | 4 | 9.96e-01 |
| R66 | THP1,CCL3,HD2 | HD2Y5W | 4 | 4.17e-01 |
| R66 | THP1,CCL3,HD2T6W | HD2Y5W | 4 | 8.04e-01 |
| R66 | THP1,CCL3,HD2T6D | HD2Y5W | 4 | 4.20e-01 |
| R66 | THP1,CCL3,HD2A9W | HD2Y5W | 4 | 9.99e-01 |
| R66 | THP1,CCL3,HD2L11I | HD2Y5W | 4 | 9.94e-01 |
| R66 | THP1,CCL3,HD2T12W | HD2Y5W | 4 | 1.43e-02 |
| R66 | THP1,CCL3,HD2T16C | HD2Y5W | 4 | 4.02e-01 |
| R66 | THP1,CCL3,HD2CM304 | HD2Y5W | 4 | 8.46e-02 |
| R66 | THP1,CCL3,HD2CM307 | HD2Y5W | 4 | 7.38e-02 |
| R66 | THP1,CCL3,HD2CM452 | HD2Y5W | 4 | 9.96e-01 |
| R66 | THP1,CCL3,HD2CM526 | HD2Y5W | 4 | 9.99e-01 |
| R66 | THP1,CCL3,HD2CM539 | HD2Y5W | 4 | 8.24e-01 |
| R67 | ATC | HD2Y5W | 3 | 0.00e+00 |
| R67 | ATC,CXCL10 | HD2Y5W | 3 | 1.00e+00 |
| R67 | ATC,CXCL10,HD2Y5W | HD2Y5W | 3 | NA |
| R67 | ATC,CXCL10,HD2SCR | HD2Y5W | 3 | 1.00e+00 |
| R67 | ATC,CXCL10,HD2 | HD2Y5W | 3 | 1.00e+00 |
| R67 | ATC,CXCL10,HD2T6W | HD2Y5W | 3 | 9.07e-01 |
| R67 | ATC,CXCL10,HD2T6D | HD2Y5W | 3 | 9.74e-01 |
| R67 | ATC,CXCL10,HD2A9W | HD2Y5W | 3 | 5.02e-02 |
| R67 | ATC,CXCL10,HD2L11I | HD2Y5W | 3 | 1.00e+00 |
| R67 | ATC,CXCL10,HD2T12W | HD2Y5W | 3 | 1.00e+00 |
| R67 | ATC,CXCL10,HD2T16C | HD2Y5W | 3 | 1.00e+00 |
| R67 | ATC,CXCL10,HD2CM304 | HD2Y5W | 3 | 1.00e+00 |
| R67 | ATC,CXCL10,HD2CM307 | HD2Y5W | 3 | 4.38e-03 |
| R67 | ATC,CXCL10,HD2CM452 | HD2Y5W | 3 | 5.05e-01 |
| R67 | ATC,CXCL10,HD2CM526 | HD2Y5W | 3 | 4.11e-03 |
| R67 | ATC,CXCL10,HD2CM539 | HD2Y5W | 3 | 7.72e-03 |
| R79 | THP1 | HD2Y5W | 3 | 4.09e-04 |
| R79 | THP1,CCL7 | HD2Y5W | 3 | 1.07e-04 |
| R79 | THP1,CCL7,HD2Y5W | HD2Y5W | 3 | NA |
| R79 | THP1,CCL7,HD2SCR | HD2Y5W | 3 | 1.33e-03 |
| R79 | THP1,CCL7,HD2 | HD2Y5W | 3 | 9.67e-01 |
| R79 | THP1,CCL7,HD2T6W | HD2Y5W | 3 | 9.95e-01 |
| R79 | THP1,CCL7,HD2T6D | HD2Y5W | 3 | 9.96e-01 |
| R79 | THP1,CCL7,HD2L11I | HD2Y5W | 3 | 4.56e-01 |
| R79 | THP1,CCL7,HD2T12W | HD2Y5W | 3 | 4.55e-01 |
| R79 | THP1,CCL7,HD2T16C | HD2Y5W | 3 | 7.22e-01 |
| R79 | THP1,CCL7,HD2CM304 | HD2Y5W | 3 | 4.48e-01 |
| R79 | THP1,CCL7,HD2CM307 | HD2Y5W | 3 | 2.03e-03 |
| R79 | THP1,CCL7,HD2CM452 | HD2Y5W | 3 | 1.00e+00 |
| R79 | THP1,CCL7,HD2CM526 | HD2Y5W | 3 | 1.00e+00 |
| R79 | THP1,CCL7,HD2CM539 | HD2Y5W | 3 | 2.05e-02 |
| R86 | THP1 | HD2Y5W | 3 | 2.71e-01 |
| R86 | THP1,CCL8 | HD2Y5W | 3 | 1.98e-03 |
| R86 | THP1,CCL8,HD2Y5W | HD2Y5W | 3 | NA |
| R86 | THP1,CCL8,HD2SCR | HD2Y5W | 3 | 3.39e-03 |
| R86 | THP1,CCL8,HD2 | HD2Y5W | 3 | 2.71e-02 |
| R86 | THP1,CCL8,HD2T6W | HD2Y5W | 3 | 9.27e-01 |
| R86 | THP1,CCL8,HD2T6D | HD2Y5W | 3 | 8.48e-01 |
| R86 | THP1,CCL8,HD2L11I | HD2Y5W | 3 | 1.48e-01 |
| R86 | THP1,CCL8,HD2T12W | HD2Y5W | 3 | 1.83e-01 |
| R86 | THP1,CCL8,HD2T16C | HD2Y5W | 3 | 3.21e-03 |
| R86 | THP1,CCL8,HD2CM304 | HD2Y5W | 3 | 6.17e-02 |
| R86 | THP1,CCL8,HD2CM307 | HD2Y5W | 3 | 1.00e+00 |
| R86 | THP1,CCL8,HD2CM452 | HD2Y5W | 3 | 1.32e-01 |
| R86 | THP1,CCL8,HD2CM526 | HD2Y5W | 3 | 6.76e-01 |
| R86 | THP1,CCL8,HD2CM539 | HD2Y5W | 3 | 9.17e-01 |
| R94 | THP1 | HD2 | 3 | 4.03e-01 |
| R94 | THP1,CCL8 | HD2 | 3 | 6.24e-04 |
| R94 | THP1,CCL8,HD2 | HD2 | 3 | NA |
| R94 | THP1,CCL8,HD2SCR | HD2 | 3 | 5.70e-05 |
| R94 | THP1,CCL8,HD2Y5W | HD2 | 3 | 1.22e-02 |
| R94 | THP1,CCL8,HD2CM307 | HD2 | 3 | 6.04e-01 |
| R94 | THP1,CCL8,HD2CM3085 | HD2 | 3 | 3.77e-04 |
| R95 | THP1 | HD2 | 3 | 5.38e-03 |
| R95 | THP1,CCL7 | HD2 | 3 | 2.17e-04 |
| R95 | THP1,CCL7,HD2 | HD2 | 3 | NA |
| R95 | THP1,CCL7,HD2SCR | HD2 | 3 | 6.96e-04 |
| R95 | THP1,CCL7,HD2Y5W | HD2 | 3 | 5.15e-01 |
| R95 | THP1,CCL7,HD2CM307 | HD2 | 3 | 1.12e-02 |
| R95 | THP1,CCL7,HD2CM3085 | HD2 | 3 | 7.88e-01 |
| R97 | ATC | HD2SCR | 3 | 0.00e+00 |
| R97 | ATC,INS | HD2SCR | 3 | 2.77e-01 |
| R97 | ATC,INS,HD2SCR | HD2SCR | 3 | NA |
| R97 | ATC,INS,HD2 | HD2SCR | 3 | 0.00e+00 |
| R97 | ATC,INS,HD2Y5W | HD2SCR | 3 | 0.00e+00 |
| R97 | ATC,INS,HD2T6W | HD2SCR | 3 | 0.00e+00 |
| R97 | ATC,INS,HD2T6D | HD2SCR | 3 | 3.50e-05 |
| R97 | ATC,INS,HD2L11I | HD2SCR | 3 | 0.00e+00 |
| R97 | ATC,INS,HD2T12W | HD2SCR | 3 | 8.00e-06 |
| R97 | ATC,INS,HD2T16C | HD2SCR | 3 | 0.00e+00 |
| R97 | ATC,INS,HD2CM304 | HD2SCR | 3 | 0.00e+00 |
| R97 | ATC,INS,HD2CM307 | HD2SCR | 3 | 0.00e+00 |
| R97 | ATC,INS,HD2CM452 | HD2SCR | 3 | 0.00e+00 |
| R97 | ATC,INS,HD2CM526 | HD2SCR | 3 | 1.50e-05 |
| R97 | ATC,INS,HD2CM539 | HD2SCR | 3 | 0.00e+00 |
| R97 | ATC,INS,EVA4 | HD2SCR | 3 | 1.00e-06 |
| R105 | Granulocytes | HD2SCR | 3 | 2.40e-04 |
| R105 | Granulocytes,RA | HD2SCR | 3 | 6.59e-01 |
| R105 | Granulocytes,RA,HD2SCR | HD2SCR | 3 | NA |
| R105 | Granulocytes,RA,HD2 | HD2SCR | 3 | 9.78e-01 |
| R105 | Granulocytes,RA,HD2Y5W | HD2SCR | 3 | 9.51e-02 |
| R105 | Granulocytes,RA,HD2T6W | HD2SCR | 3 | 5.41e-01 |
| R105 | Granulocytes,RA,HD2T6D | HD2SCR | 3 | 9.80e-05 |
| R105 | Granulocytes,RA,HD2L11I | HD2SCR | 3 | 1.00e+00 |
| R105 | Granulocytes,RA,HD2T12W | HD2SCR | 3 | 7.67e-01 |
| R105 | Granulocytes,RA,HD2T16C | HD2SCR | 3 | 1.24e-01 |
| R105 | Granulocytes,RA,HD2CM304 | HD2SCR | 3 | 1.27e-02 |
| R105 | Granulocytes,RA,HD2CM307 | HD2SCR | 3 | 8.96e-01 |
| R105 | Granulocytes,RA,HD2CM452 | HD2SCR | 3 | 1.00e+00 |
| R105 | Granulocytes,RA,HD2CM526 | HD2SCR | 3 | 1.00e+00 |
| R105 | Granulocytes,RA,HD2CM539 | HD2SCR | 3 | 9.99e-01 |
| R105 | Granulocytes,RA,EVA4 | HD2SCR | 3 | 5.17e-01 |
| R106 | Monocytes | HD2SCR | 3 | 5.45e-03 |
| R106 | Monocytes,RA | HD2SCR | 3 | 1.00e+00 |
| R106 | Monocytes,RA,HD2SCR | HD2SCR | 3 | NA |
| R106 | Monocytes,RA,HD2 | HD2SCR | 3 | 1.00e+00 |
| R106 | Monocytes,RA,HD2Y5W | HD2SCR | 3 | 9.98e-01 |
| R106 | Monocytes,RA,HD2T6W | HD2SCR | 3 | 1.00e+00 |
| R106 | Monocytes,RA,HD2T6D | HD2SCR | 3 | 9.95e-01 |
| R106 | Monocytes,RA,HD2L11I | HD2SCR | 3 | 8.34e-01 |
| R106 | Monocytes,RA,HD2T12W | HD2SCR | 3 | 7.60e-01 |
| R106 | Monocytes,RA,HD2T16C | HD2SCR | 3 | 5.74e-01 |
| R106 | Monocytes,RA,HD2CM304 | HD2SCR | 3 | 5.31e-01 |
| R106 | Monocytes,RA,HD2CM307 | HD2SCR | 3 | 3.85e-01 |
| R106 | Monocytes,RA,HD2CM452 | HD2SCR | 3 | 9.23e-01 |
| R106 | Monocytes,RA,HD2CM526 | HD2SCR | 3 | 1.00e+00 |
| R106 | Monocytes,RA,HD2CM539 | HD2SCR | 3 | 6.07e-01 |
| R106 | Monocytes,RA,EVA4 | HD2SCR | 3 | 7.62e-01 |
| R107 | Lymphocytes | HD2SCR | 3 | 1.60e-05 |
| R107 | Lymphocytes,RA | HD2SCR | 3 | 2.88e-01 |
| R107 | Lymphocytes,RA,HD2SCR | HD2SCR | 3 | NA |
| R107 | Lymphocytes,RA,HD2 | HD2SCR | 3 | 9.95e-01 |
| R107 | Lymphocytes,RA,HD2Y5W | HD2SCR | 3 | 1.00e+00 |
| R107 | Lymphocytes,RA,HD2T6W | HD2SCR | 3 | 5.49e-01 |
| R107 | Lymphocytes,RA,HD2T6D | HD2SCR | 3 | 9.81e-01 |
| R107 | Lymphocytes,RA,HD2L11I | HD2SCR | 3 | 7.03e-01 |
| R107 | Lymphocytes,RA,HD2T12W | HD2SCR | 3 | 5.34e-01 |
| R107 | Lymphocytes,RA,HD2T16C | HD2SCR | 3 | 7.39e-02 |
| R107 | Lymphocytes,RA,HD2CM304 | HD2SCR | 3 | 2.09e-03 |
| R107 | Lymphocytes,RA,HD2CM307 | HD2SCR | 3 | 1.04e-01 |
| R107 | Lymphocytes,RA,HD2CM452 | HD2SCR | 3 | 8.24e-03 |
| R107 | Lymphocytes,RA,HD2CM526 | HD2SCR | 3 | 1.00e+00 |
| R107 | Lymphocytes,RA,HD2CM539 | HD2SCR | 3 | 1.00e+00 |
| R107 | Lymphocytes,RA,EVA4 | HD2SCR | 3 | 6.26e-01 |
| R108 | Monocytes | HD2SCR | 3 | 4.00e-06 |
| R108 | Monocytes,ATHERO | HD2SCR | 3 | 9.94e-01 |
| R108 | Monocytes,ATHERO,HD2SCR | HD2SCR | 3 | NA |
| R108 | Monocytes,ATHERO,HD2 | HD2SCR | 3 | 9.99e-01 |
| R108 | Monocytes,ATHERO,HD2Y5W | HD2SCR | 3 | 6.44e-01 |
| R108 | Monocytes,ATHERO,HD2T6W | HD2SCR | 3 | 1.00e+00 |
| R108 | Monocytes,ATHERO,HD2T6D | HD2SCR | 3 | 1.00e+00 |
| R108 | Monocytes,ATHERO,HD2A9W | HD2SCR | 3 | 9.99e-01 |
| R108 | Monocytes,ATHERO,HD2L11I | HD2SCR | 3 | 1.00e+00 |
| R108 | Monocytes,ATHERO,HD2T12W | HD2SCR | 3 | 6.75e-01 |
| R108 | Monocytes,ATHERO,HD2T16C | HD2SCR | 3 | 9.91e-01 |
| R108 | Monocytes,ATHERO,HD2CM304 | HD2SCR | 3 | 4.05e-01 |
| R108 | Monocytes,ATHERO,HD2CM307 | HD2SCR | 3 | 9.98e-01 |
| R108 | Monocytes,ATHERO,HD2CM452 | HD2SCR | 3 | 3.98e-01 |
| R108 | Monocytes,ATHERO,HD2CM526 | HD2SCR | 3 | 6.84e-01 |
| R108 | Monocytes,ATHERO,HD2CM539 | HD2SCR | 3 | 3.46e-01 |
| R108 | Monocytes,ATHERO,EVA4 | HD2SCR | 3 | 2.99e-03 |
| R109 | Granulocytes | HD2SCR | 3 | 3.53e-04 |
| R109 | Granulocytes,ATHERO | HD2SCR | 3 | 1.00e+00 |
| R109 | Granulocytes,ATHERO,HD2SCR | HD2SCR | 3 | NA |
| R109 | Granulocytes,ATHERO,HD2 | HD2SCR | 3 | 9.45e-01 |
| R109 | Granulocytes,ATHERO,HD2Y5W | HD2SCR | 3 | 1.33e-01 |
| R109 | Granulocytes,ATHERO,HD2T6W | HD2SCR | 3 | 9.79e-01 |
| R109 | Granulocytes,ATHERO,HD2T6D | HD2SCR | 3 | 1.00e+00 |
| R109 | Granulocytes,ATHERO,HD2A9W | HD2SCR | 3 | 9.79e-02 |
| R109 | Granulocytes,ATHERO,HD2L11I | HD2SCR | 3 | 9.99e-01 |
| R109 | Granulocytes,ATHERO,HD2T12W | HD2SCR | 3 | 1.00e+00 |
| R109 | Granulocytes,ATHERO,HD2T16C | HD2SCR | 3 | 9.71e-01 |
| R109 | Granulocytes,ATHERO,HD2CM304 | HD2SCR | 3 | 6.72e-01 |
| R109 | Granulocytes,ATHERO,HD2CM307 | HD2SCR | 3 | 2.60e-01 |
| R109 | Granulocytes,ATHERO,HD2CM452 | HD2SCR | 3 | 1.00e+00 |
| R109 | Granulocytes,ATHERO,HD2CM526 | HD2SCR | 3 | 1.46e-01 |
| R109 | Granulocytes,ATHERO,HD2CM539 | HD2SCR | 3 | 3.91e-01 |
| R109 | Granulocytes,ATHERO,EVA4 | HD2SCR | 3 | 9.08e-02 |
| R110 | Lymphocytes | HD2SCR | 3 | 6.00e-06 |
| R110 | Lymphocytes,ATHERO | HD2SCR | 3 | 1.00e+00 |
| R110 | Lymphocytes,ATHERO,HD2SCR | HD2SCR | 3 | NA |
| R110 | Lymphocytes,ATHERO,HD2 | HD2SCR | 3 | 3.59e-02 |
| R110 | Lymphocytes,ATHERO,HD2Y5W | HD2SCR | 3 | 1.24e-01 |
| R110 | Lymphocytes,ATHERO,HD2T6W | HD2SCR | 3 | 3.24e-01 |
| R110 | Lymphocytes,ATHERO,HD2T6D | HD2SCR | 3 | 9.99e-01 |
| R110 | Lymphocytes,ATHERO,HD2A9W | HD2SCR | 3 | 5.49e-02 |
| R110 | Lymphocytes,ATHERO,HD2L11I | HD2SCR | 3 | 1.34e-01 |
| R110 | Lymphocytes,ATHERO,HD2T12W | HD2SCR | 3 | 9.03e-01 |
| R110 | Lymphocytes,ATHERO,HD2T16C | HD2SCR | 3 | 1.26e-02 |
| R110 | Lymphocytes,ATHERO,HD2CM304 | HD2SCR | 3 | 1.57e-03 |
| R110 | Lymphocytes,ATHERO,HD2CM307 | HD2SCR | 3 | 5.70e-02 |
| R110 | Lymphocytes,ATHERO,HD2CM452 | HD2SCR | 3 | 2.75e-03 |
| R110 | Lymphocytes,ATHERO,HD2CM526 | HD2SCR | 3 | 7.16e-01 |
| R110 | Lymphocytes,ATHERO,HD2CM539 | HD2SCR | 3 | 3.69e-01 |
| R110 | Lymphocytes,ATHERO,EVA4 | HD2SCR | 3 | 1.51e-04 |
| R124 | ATC | HD2SCR | 3 | 0.00e+00 |
| R124 | ATC,ATHERO | HD2SCR | 3 | 1.00e+00 |
| R124 | ATC,ATHERO,HD2SCR | HD2SCR | 3 | NA |
| R124 | ATC,ATHERO,HD2 | HD2SCR | 3 | 0.00e+00 |
| R124 | ATC,ATHERO,HD2Y5W | HD2SCR | 3 | 8.51e-04 |
| R124 | ATC,ATHERO,HD2T6W | HD2SCR | 3 | 0.00e+00 |
| R124 | ATC,ATHERO,HD2T6D | HD2SCR | 3 | 1.90e-05 |
| R124 | ATC,ATHERO,HD2A9W | HD2SCR | 3 | 0.00e+00 |
| R124 | ATC,ATHERO,HD2L11I | HD2SCR | 3 | 0.00e+00 |
| R124 | ATC,ATHERO,HD2T12W | HD2SCR | 3 | 0.00e+00 |
| R124 | ATC,ATHERO,HD2T16C | HD2SCR | 3 | 0.00e+00 |
| R124 | ATC,ATHERO,HD2CM304 | HD2SCR | 3 | 0.00e+00 |
| R124 | ATC,ATHERO,HD2CM307 | HD2SCR | 3 | 0.00e+00 |
| R124 | ATC,ATHERO,HD2CM452 | HD2SCR | 3 | 0.00e+00 |
| R124 | ATC,ATHERO,HD2CM526 | HD2SCR | 3 | 5.40e-05 |
| R124 | ATC,ATHERO,HD2CM539 | HD2SCR | 3 | 0.00e+00 |
| R124 | ATC,ATHERO,EVA4 | HD2SCR | 3 | 0.00e+00 |
| R135 | ATC,RA | HD2SCR | 3 | 1.93e-04 |
| R135 | ATC,RA,HD2SCR | HD2SCR | 3 | NA |
| R135 | ATC,RA,HD2 | HD2SCR | 3 | 6.92e-01 |
| R135 | ATC,RA,HD2Y5W | HD2SCR | 3 | 0.00e+00 |
| R135 | ATC,RA,HD2T6W | HD2SCR | 3 | 4.27e-02 |
| R135 | ATC,RA,HD2T6D | HD2SCR | 3 | 9.44e-01 |
| R135 | ATC,RA,HD2L11I | HD2SCR | 3 | 8.08e-02 |
| R135 | ATC,RA,HD2T12W | HD2SCR | 3 | 3.73e-01 |
| R135 | ATC,RA,HD2CM304 | HD2SCR | 3 | 0.00e+00 |
| R135 | ATC,RA,HD2CM307 | HD2SCR | 3 | 7.00e-06 |
| R135 | ATC,RA,HD2CM452 | HD2SCR | 3 | 0.00e+00 |
| R135 | ATC,RA,HD2CM526 | HD2SCR | 3 | 7.08e-01 |
| R135 | ATC,RA,HD2CM539 | HD2SCR | 3 | 2.92e-03 |
| R135 | ATC,RA,EVA4 | HD2SCR | 3 | 0.00e+00 |
| R137 | Granulocytes | HD2SCR | 3 | 8.60e-05 |
| R137 | Granulocytes,INS | HD2SCR | 3 | 8.10e-01 |
| R137 | Granulocytes,INS,HD2SCR | HD2SCR | 3 | NA |
| R137 | Granulocytes,INS,HD2 | HD2SCR | 3 | 1.51e-01 |
| R137 | Granulocytes,INS,HD2Y5W | HD2SCR | 3 | 8.00e-01 |
| R137 | Granulocytes,INS,HD2T6W | HD2SCR | 3 | 1.00e+00 |
| R137 | Granulocytes,INS,HD2T6D | HD2SCR | 3 | 1.00e+00 |
| R137 | Granulocytes,INS,HD2L11I | HD2SCR | 3 | 9.99e-01 |
| R137 | Granulocytes,INS,HD2T12W | HD2SCR | 3 | 1.00e+00 |
| R137 | Granulocytes,INS,HD2T16C | HD2SCR | 3 | 9.12e-01 |
| R137 | Granulocytes,INS,HD2CM304 | HD2SCR | 3 | 1.00e+00 |
| R137 | Granulocytes,INS,HD2CM307 | HD2SCR | 3 | 9.97e-01 |
| R137 | Granulocytes,INS,HD2CM452 | HD2SCR | 3 | 1.00e+00 |
| R137 | Granulocytes,INS,HD2CM526 | HD2SCR | 3 | 1.00e+00 |
| R137 | Granulocytes,INS,HD2CM539 | HD2SCR | 3 | 1.00e+00 |
| R137 | Granulocytes,INS,EVA4 | HD2SCR | 3 | 1.00e+00 |
| R138 | Monocytes | HD2SCR | 3 | 2.80e-05 |
| R138 | Monocytes,INS | HD2SCR | 3 | 8.06e-01 |
| R138 | Monocytes,INS,HD2SCR | HD2SCR | 3 | NA |
| R138 | Monocytes,INS,HD2 | HD2SCR | 3 | 9.94e-01 |
| R138 | Monocytes,INS,HD2Y5W | HD2SCR | 3 | 4.03e-02 |
| R138 | Monocytes,INS,HD2T6W | HD2SCR | 3 | 1.48e-01 |
| R138 | Monocytes,INS,HD2T6D | HD2SCR | 3 | 1.67e-02 |
| R138 | Monocytes,INS,HD2L11I | HD2SCR | 3 | 6.67e-01 |
| R138 | Monocytes,INS,HD2T12W | HD2SCR | 3 | 5.37e-01 |
| R138 | Monocytes,INS,HD2T16C | HD2SCR | 3 | 1.00e+00 |
| R138 | Monocytes,INS,HD2CM304 | HD2SCR | 3 | 5.80e-02 |
| R138 | Monocytes,INS,HD2CM307 | HD2SCR | 3 | 1.44e-03 |
| R138 | Monocytes,INS,HD2CM452 | HD2SCR | 3 | 8.98e-02 |
| R138 | Monocytes,INS,HD2CM526 | HD2SCR | 3 | 2.04e-01 |
| R138 | Monocytes,INS,HD2CM539 | HD2SCR | 3 | 2.47e-03 |
| R138 | Monocytes,INS,EVA4 | HD2SCR | 3 | 8.10e-05 |
| R139 | Lymphocytes | HD2SCR | 3 | 4.23e-02 |
| R139 | Lymphocytes,INS | HD2SCR | 3 | 9.92e-01 |
| R139 | Lymphocytes,INS,HD2SCR | HD2SCR | 3 | NA |
| R139 | Lymphocytes,INS,HD2 | HD2SCR | 3 | 1.00e+00 |
| R139 | Lymphocytes,INS,HD2Y5W | HD2SCR | 3 | 1.00e+00 |
| R139 | Lymphocytes,INS,HD2T6W | HD2SCR | 3 | 9.89e-01 |
| R139 | Lymphocytes,INS,HD2T6D | HD2SCR | 3 | 1.00e+00 |
| R139 | Lymphocytes,INS,HD2L11I | HD2SCR | 3 | 1.00e+00 |
| R139 | Lymphocytes,INS,HD2T12W | HD2SCR | 3 | 9.99e-01 |
| R139 | Lymphocytes,INS,HD2T16C | HD2SCR | 3 | 6.48e-01 |
| R139 | Lymphocytes,INS,HD2CM304 | HD2SCR | 3 | 5.78e-01 |
| R139 | Lymphocytes,INS,HD2CM307 | HD2SCR | 3 | 7.82e-01 |
| R139 | Lymphocytes,INS,HD2CM452 | HD2SCR | 3 | 9.16e-01 |
| R139 | Lymphocytes,INS,HD2CM526 | HD2SCR | 3 | 1.00e+00 |
| R139 | Lymphocytes,INS,HD2CM539 | HD2SCR | 3 | 9.84e-01 |
| R139 | Lymphocytes,INS,EVA4 | HD2SCR | 3 | 1.00e+00 |
| R148 | THP1 | HD2Y5W | 3 | 0.00e+00 |
| R148 | THP1,CCL23 | HD2Y5W | 3 | 6.87e-01 |
| R148 | THP1,CCL23,HD2Y5W | HD2Y5W | 3 | NA |
| R148 | THP1,CCL23,HD2SCR | HD2Y5W | 3 | 5.15e-01 |
| R148 | THP1,CCL23,HD2 | HD2Y5W | 3 | 8.27e-01 |
| R148 | THP1,CCL23,HD2T6W | HD2Y5W | 3 | 8.90e-01 |
| R148 | THP1,CCL23,HD2T6D | HD2Y5W | 3 | 1.49e-01 |
| R148 | THP1,CCL23,HD2A9W | HD2Y5W | 3 | 1.00e+00 |
| R148 | THP1,CCL23,HD2L11I | HD2Y5W | 3 | 4.06e-01 |
| R148 | THP1,CCL23,HD2T12W | HD2Y5W | 3 | 8.31e-01 |
| R148 | THP1,CCL23,HD2T16C | HD2Y5W | 3 | 1.00e+00 |
| R148 | THP1,CCL23,HD2CM304 | HD2Y5W | 3 | 3.55e-01 |
| R148 | THP1,CCL23,HD2CM307 | HD2Y5W | 3 | 5.26e-01 |
| R148 | THP1,CCL23,HD2CM452 | HD2Y5W | 3 | 9.56e-01 |
| R148 | THP1,CCL23,HD2CM526 | HD2Y5W | 3 | 5.55e-01 |
| R148 | THP1,CCL23,HD2CM539 | HD2Y5W | 3 | 4.22e-02 |
| R86a | THP1 | HD2 | 3 | 1.10e-04 |
| R86a | THP1,CCL8 | HD2 | 3 | 9.55e-01 |
| R86a | THP1,CCL8,HD2 | HD2 | 3 | NA |
| R86a | THP1,CCL8,HD2SCR | HD2 | 3 | 9.89e-01 |
| R86a | THP1,CCL8,HD2Y5W | HD2 | 3 | 2.71e-02 |
| R86a | THP1,CCL8,HD2T6W | HD2 | 3 | 2.71e-01 |
| R86a | THP1,CCL8,HD2T6D | HD2 | 3 | 1.04e-03 |
| R86a | THP1,CCL8,HD2L11I | HD2 | 3 | 9.95e-01 |
| R86a | THP1,CCL8,HD2T12W | HD2 | 3 | 9.84e-01 |
| R86a | THP1,CCL8,HD2T16C | HD2 | 3 | 9.89e-01 |
| R86a | THP1,CCL8,HD2CM304 | HD2 | 3 | 1.00e+00 |
| R86a | THP1,CCL8,HD2CM307 | HD2 | 3 | 8.72e-03 |
| R86a | THP1,CCL8,HD2CM452 | HD2 | 3 | 9.97e-01 |
| R86a | THP1,CCL8,HD2CM526 | HD2 | 3 | 5.19e-01 |
| R86a | THP1,CCL8,HD2CM539 | HD2 | 3 | 1.57e-03 |
